# GWAS provides new insights into the genetic mechanisms of phytochemicals production and red skin colour in apple

**DOI:** 10.1093/hr/uhac218

**Published:** 2022-09-26

**Authors:** Satish Kumar, Claire Molloy, Martin Hunt, Cecilia Hong Deng, Claudia Wiedow, Christelle Andre, Andrew Dare, Tony McGhie

**Affiliations:** The New Zealand Institute for Plant and Food Research Limited, Hawke’s Bay Research Centre, Havelock North 4130, New Zealand; The New Zealand Institute for Plant and Food Research Limited, Hawke’s Bay Research Centre, Havelock North 4130, New Zealand; The New Zealand Institute for Plant and Food Research Limited, Palmerston North Research Centre, Palmerston North 4410, New Zealand; The New Zealand Institute for Plant and Food Research Limited, Mount Albert Research Centre, Auckland 1025, New Zealand; The New Zealand Institute for Plant and Food Research Limited, Palmerston North Research Centre, Palmerston North 4410, New Zealand; The New Zealand Institute for Plant and Food Research Limited, Mount Albert Research Centre, Auckland 1025, New Zealand; The New Zealand Institute for Plant and Food Research Limited, Mount Albert Research Centre, Auckland 1025, New Zealand; The New Zealand Institute for Plant and Food Research Limited, Palmerston North Research Centre, Palmerston North 4410, New Zealand

## Abstract

Understanding the genetic architecture of apple phytochemicals, and their interplay with conventional selection traits, is critical for the development of new apple cultivars with enhanced health benefits. Apple accessions (n = 344) used for this genome-wide association study (GWAS) represented the wide diversity of metabolic profiles in the domesticated and wild *Malus* genepools. Fruit samples were phenotyped for 34 metabolites, including a stable vitamin C glycoside “ascorbic acid 2-β-glucoside” (AA-2βG), and the accessions were genotyped using the Apple 20 K SNP Array. Several fruit quality traits, including red skin over-colour (OCOL), were also assessed. Wild *Malus* accessions showed at least 2-fold higher average content of several metabolites (e.g. ascorbic acid, chlorogenic acid, phloridzin, and trilobatin) than *Malus domestica* accessions. Several new genomic regions and potential candidate genes underpinning the genetic diversity of apple phytochemicals were identified. The percentage of phenotypic variance explained by the best SNP ranged between 3% and 21% for the different metabolites. Novel association signals for OCOL in the syntenic regions on chromosomes 13 and 16 suggested that whole genome duplication has played a role in the evolution of apple red skin colour. Genetic correlations between phytochemicals and sensory traits were moderate. This study will assist in the selection of *Malus* accessions with specific phytochemical profiles to establish innovative genomics-based breeding strategies for the development of apple cultivars with enhanced nutritional value.

## Introduction

Apples are among the most consumed fruit worldwide, and studies have shown a clear trend for higher polyphenol content in whole apples leading to greater overall health benefits [1]. Apple polyphenols can be extracted and used as functional food materials that aid in weight management, satiety and obesity prevention. Consumption of apple polyphenol extract was found to have an inhibitory effect on the accumulation of visceral fat in humans [1]. The apple phytochemicals are widely recognised for their antioxidant activity, suggesting that apple or constituents of apple may contribute to increased total antioxidant capacity in human plasma. Various flavonoids compounds are found in apple, and studies in humans using flavonoid-containing extracts showed enhanced cognitive function and memory performance [2].

The visual characteristics (e.g. skin characteristics), eating quality (e.g. texture and flavour), and storability are among the main fruit quality traits being targeted in apple breeding programmes, but the enhancement of phytochemicals is now gaining traction to select “bio-fortified” apple cultivars. Farneti *et al*. [3] screened various accessions of *Malus domestica* and wild *Malus* species and reported that modern apples have drastically reduced polyphenol content compared with the ancestral heritage and germline cultivars. Davies *et al*. [4] found that the phenolic content in cultivated apples was 68% lower than in their wild progenitors (*Malus sieversii*), and suggested that selection against internal flesh browning and bitterness could have contributed to the lower phenolic content in commercial cultivars. There is an opportunity to utilise the diversity of wild apples to enhance the nutritional content of future cultivars.

QTL mapping studies, using relatively small bi-parental populations (ca. 150) of *M. domestica*, have identified the upper region of chromosome 16 as a potential hotspot for apple phytochemicals [7, 8]. In a genome-wide association study (GWAS) on fruit samples of 136 *M. domestica* accessions, McClure *et al*. [9] reported that the concentrations of polyphenols varied by multiple folds across cultivars, and that most polyphenols could be predicted using DNA markers. In order to capture genetic diversity across species, a recent GWAS using 124 accessions of *M. domestica* and *Malus* spp. identified marker-trait associations for phytochemicals on each linkage group [10]. Apples with higher ascorbic acid (AsA) content would not only provide additional health benefits, but would also enhance the storage and shelf-life of the fruit. The recent discovery of a more stable glycosylated version of AsA, “ascorbic acid 2-β-glucoside” (AA-2βG), in wild *Malus* species offers opportunities to develop new cultivars with all the associated health benefits of vitamin C [5]. Several studies have been conducted to identify QTL for the AsA biosynthesis [6], but apparently there are no published reports on the genetic control of AA-2βG in fruit crops.

Studies on apple phytochemicals have reported on the genetic variation [3, 11] and marker-trait associations [9], but the genetic relationships between apple fruit quality traits (especially sensory traits) and flavonoids have not been reported. The complex quantitative nature of fruit quality traits and phytochemicals can be affected by genetic and non-genetic factors, hence an understanding of the genetic interplay between these two groups of traits would be vital for parental selection and apple cultivar breeding. In the present GWAS, we used by far the largest sample set (ca. 350) of *M. domestica* and wild *Malus* species accessions to assess the diversity and genetic architecture of main bioactive compounds of apple fruit. Marker-trait association analyses were conducted, and the relationships between sensory traits and phytochemicals were also investigated to guide the development of bio-fortified apple cultivars.

## Results

### Variation of phytochemicals in apple germplasm

The targeted LC–MS analyses of fruit samples revealed the presence of 34 compounds in the accessions of *M. domestica* and wild species. Seasonal variation was observed for most phytochemicals for the 15 accessions assessed in both years, with between-year correlations ranging from 0.22 for fatty acids to 0.96 for dihydrochalcones, with an average of 0.65 across all 34 compounds.

While most of the 34 compounds showed substantial variation among the 344 accessions (Supplementary Figure S1), only 14 were significantly (*p* < 0.01) different between *Malus* spp. and *M. domestica* accessions (Table 1). The concentrations of AsA, chlorogenic acid, epicatechin, phloretin, phloridzin, procyanidins B2, B7 and C1 were significantly higher in *Malus* spp. accessions by two to three fold (Table 1). The maximum phloridzin content assessed in wild *Malus* spp. accessions was 430 μg/g FW compared with a maximum of 115 μg/g FW in *M. domestica*. Similarly, the highest chlorogenic acid (CGA) content (2746 μg/g FW) in *Malus* spp. accessions was about 2.5-fold higher than the maximum of 1104 μg/g FW in cultivated apples. The average content of triterpenoids (e.g. maslinic, betulinic, ursolic and corosolic acid) in *M. domestica* accessions was 11, 8, 60 and 21 μg/g FW, respectively, which did not differ significantly from the wild species (Table 1).

**Table 1:** Distribution of the content of phytochemicals in the fruits from *Malus* germplasm

		*Malus ×domestica*			*Malus spp*.		
Trait	Code	Mean	Maximum	Detected (%)	Mean	Maximum	Detected (%)
ascorbic acid	AsA^*^	32.5	220.5	73	76.4	325.0	87
ascorbyl 2-beta-glucoside	AA-2βG^*^	0.6	49.0	2	926.9	5765.0	67
3-hydroxyphloridzin	3-OH-Phlz^*^	1.1	7.2	69	6.4	54.4	93
3-oxo-hydroxy-urs-12-en-28-oic acid_1	3-oxo-OH^*^	21.9	146.4	100	12.8	56.3	100
annurcoic acid	AnnA^*^	75.9	437.9	100	42.6	153.8	100
betulinic acid	BetuA	8.1	31.3	99	7.6	26.4	100
catechin	Catechin	28.2	210.9	92	20.9	131.8	67
chlorogenic acid	CGA^*^	186.9	1104.0	100	434.8	2745.9	97
*cis*-4-*p*-coumaroyl quinic acid	c4pCouQA	1.5	19.4	44	1.3	17.5	13
corosolic acid	CoroA	21.2	97.5	100	16.5	56.0	100
epicatechin	epiCat^*^	120.1	960.4	100	278.2	1567.6	100
euscaphic acid	EscaA	25.7	234.3	100	31.6	171.3	100
linoleic acid	LinoA	11.3	83.4	97	17.6	126.4	100
malic acid	MA^*^	8682.0	17718.1	100	11504.3	19901.1	100
maslinic acid	MasA	11.9	87.4	100	11.6	49.2	100
oleic acid	OA	12.4	80.5	88	12.5	181.8	43
phloretin-2’-*O*-xyloglucoside	phloretin^*^	22.2	156.9	100	95.3	303.1	97
phloridzin	phloridzin^*^	19.2	115.5	100	60.6	430.4	100
pomolic acid	PomoA^*^	21.3	79.7	100	15.2	36.0	100
pomaceic acid	PomaA	5.1	260.8	66	2.0	22.7	60
procyanidin B1	ProCy_B1	38.4	202.0	92	42.0	140.2	87
procyanidin B2	ProCy_B2^*^	155.7	895.3	100	536.8	2284.4	100
procyanidin B5	ProCy_B5	6.4	41.0	83	10.9	45.0	60
procyanidin B7	ProCy_B7^*^	14.4	70.1	96	39.3	167.7	93
procyanidin C1	ProCy_C1^*^	80.7	514.0	99	226.7	1074.7	93
quercetin 3-arabinopyranoside	Q-arapy	35.6	193.4	100	35.9	153.6	100
quercetin 3-galactoside	Q-gal	57.8	408.4	100	60.6	224.2	100
quercetin 3-glucoside	Q-glu	20.0	116.9	100	30.0	107.9	100
quercetin 3-rhamnoside	Q-rha	32.9	152.5	100	34.9	290.1	100
quercetin 3-rutinoside	Q-rut	8.3	58.4	95	11.2	60.4	93
quercetin 3-xyloside	Q-xyl	18.7	120.5	100	18.9	110.9	100
*trans*-4-*p*-coumaroyl quinic acid	t4pCouQA	47.5	571.9	100	48.2	287.6	83
*trans*-5-*p*-coumaroyl quinic acid	t5pCouQA	8.5	141.3	68	6.3	37.6	47
ursolic acid	UA	59.6	92.7	99	58.1	114.1	100

^*^Significantly (*p* < 0.01) different between the two genepools

**Figure 2:**
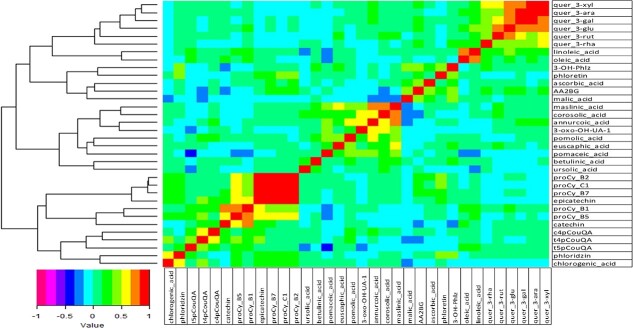
Heat map showing genetic correlations among all pairs of 34 metabolites measured across 344 apple accessions.

**Figure 1:**
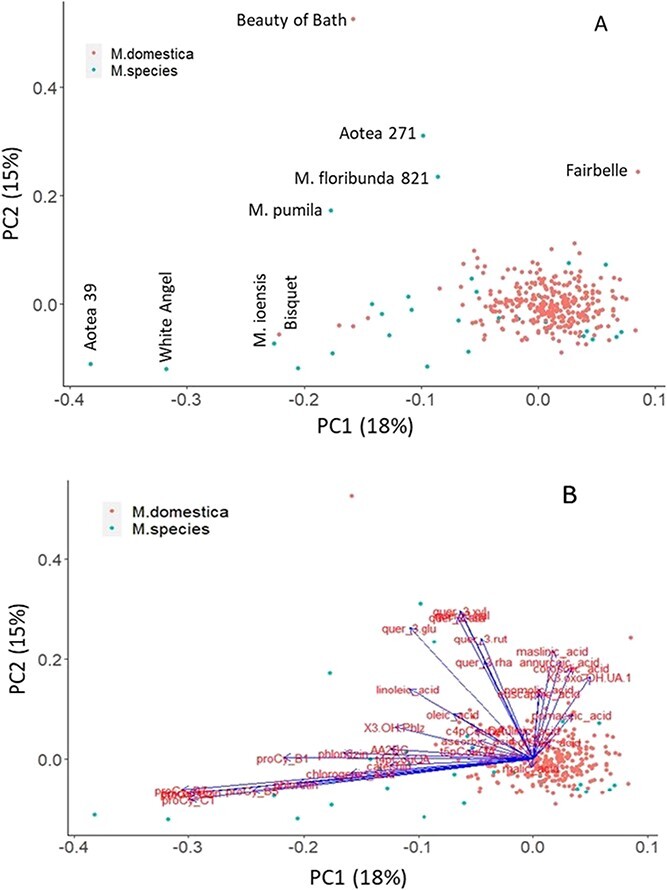
Principal component (PC) analysis (A) and loading projection (B) plots of the phytochemical compounds assessed in the *Malus domestica* and *Malus* spp. accessions.

AA-2βG was detected in two-thirds of wild species accessions, with an average of 927 μg/g FW. On the other hand, only about 2% of *M. domestica* accessions displayed AA-2βG with a very small average content (< 1.0 μg/g FW). The average content of malic acid (MA) in *M. domestica* accessions was 8682 μg/g FW, which was significantly lower compared with wild accessions (11504.3 μg/g FW) (Table 1). Trilobatin was detected in the fruit of 9 out of 31 *Malus* species accessions, and the content in “*Malus floribunda* 821” (33.1 μg/g FW) and *Malus sieboldii* “Snowbright” (29.9 μg/g FW) was about four times higher than in the *M. trilobata* accession (7.93 μg/g FW). Trilobatin was not considered for GWA analysis due to the low detection frequency. The phytochemicals-based clustering (Fig. 1A) of domesticated and wild species accessions overlapped as many of the compounds were not significantly different between the two genepools. The PCA loadings plot (Fig. 1B) suggested that the accessions of wild species were characterised by a higher content of various compounds, including procyanidin B2, procyanidin B7, procyanidin C1, phloretin, epicatechin, and chlorogenic acid – supporting the results described in Table 1.

Genomic heritability (*h*^2^) estimates suggested that most of the phytochemicals in this study have high heritability (Supplementary Figure S2). Pomaceic acid, t5pCouQA, ursolic acid and 3-hydroxyphloridzin were the least heritable (*h*^2^ < 0.20) traits, while quercetin glycosides, chlorogenic acid, phloridzin and epicatechin were among the highly heritable (*h*^2^ > 0.50) traits. AA-2βG was more heritable than AsA (0.43 versus 0.20). Flavour intensity (FINT) was the least heritable (*h*^2^ = 0.14) and the OCOL was the most heritable (*h*^2^ = 0.82) trait among the fruit quality traits (Supplementary Figure S2).

### Genetic correlations among phytochemicals and fruit quality traits

The degree of the correlations among all pairs of phytochemicals are illustrated in Fig. 2 and Supplementary Figure S3. Significant (*p* < 0.001) positive correlations among epicatechin and the procyanidins were observed. The correlations among the six quercetin compounds varied between 0.40 and 0.90 (*p* < 0.001). Although several pairs of compounds were negatively correlated, only few pairs (e.g. corosolic acid and MA; trans-5-p-coumaroyl quinic acid and pomaceic acid) were found to be significant (*p* < 0.001). Maslinic and corosolic acids showed strong positive correlation with several other compounds (e.g. annurcoic acid and 3-oxo-hydroxy-urs-12-en-28-oic acid_1). The correlation (ca. 0.40) between AsA and AA-2βG was significant, and both of these compounds were also significantly correlated with MA (Fig. 2). The clusters displayed by the dendrogram showed that the flavanols (procyanidins, catechin, and epicatechin) and flavonols (*quercetin* compounds) *clustered separately*. Similarly, several triterpene acids, such as pomaceic, annurcoic, *euscaphic*, pomolic, maslinic, betulinic and ursolic *acids also clustered together (**Fig. 2**).*

The magnitude of correlation between phytochemicals and fruit quality traits ranged between −0.35 and 0.35 (Supplementary Figure S4), and most of these were non-significant. Noteworthy relationships included a significant negative correlation between AsA and fruit browning disorder (FBD; *r* = −0.30, *p* = 8.6E-09), and between trans-4-p-coumaroyl quinic acid and eating quality (EQUA; *r* = −0.34, *p* = 5.4E-11). Not surprisingly, MA content was positively correlated with sourness (SOUR; *r* = 0.32, *p* = 1.2E-09) and flavour intensity (FINT; *r* = 0.25, *p* = 2.6E-06). Fruit skin colour (OCOL) was significantly positively correlated with ursolic acid content (*r* = 0.36, *p* = 2.6E-12). Among all the phytochemicals assessed in this study, corosolic acid showed the highest positive correlation with the fruit texture traits (FIRM: 0.29, *p* = 6.8E-08; CRISP: 0.31, *p* = 2.7E-09) (Supplementary Figure S4).

**Figure 3:**
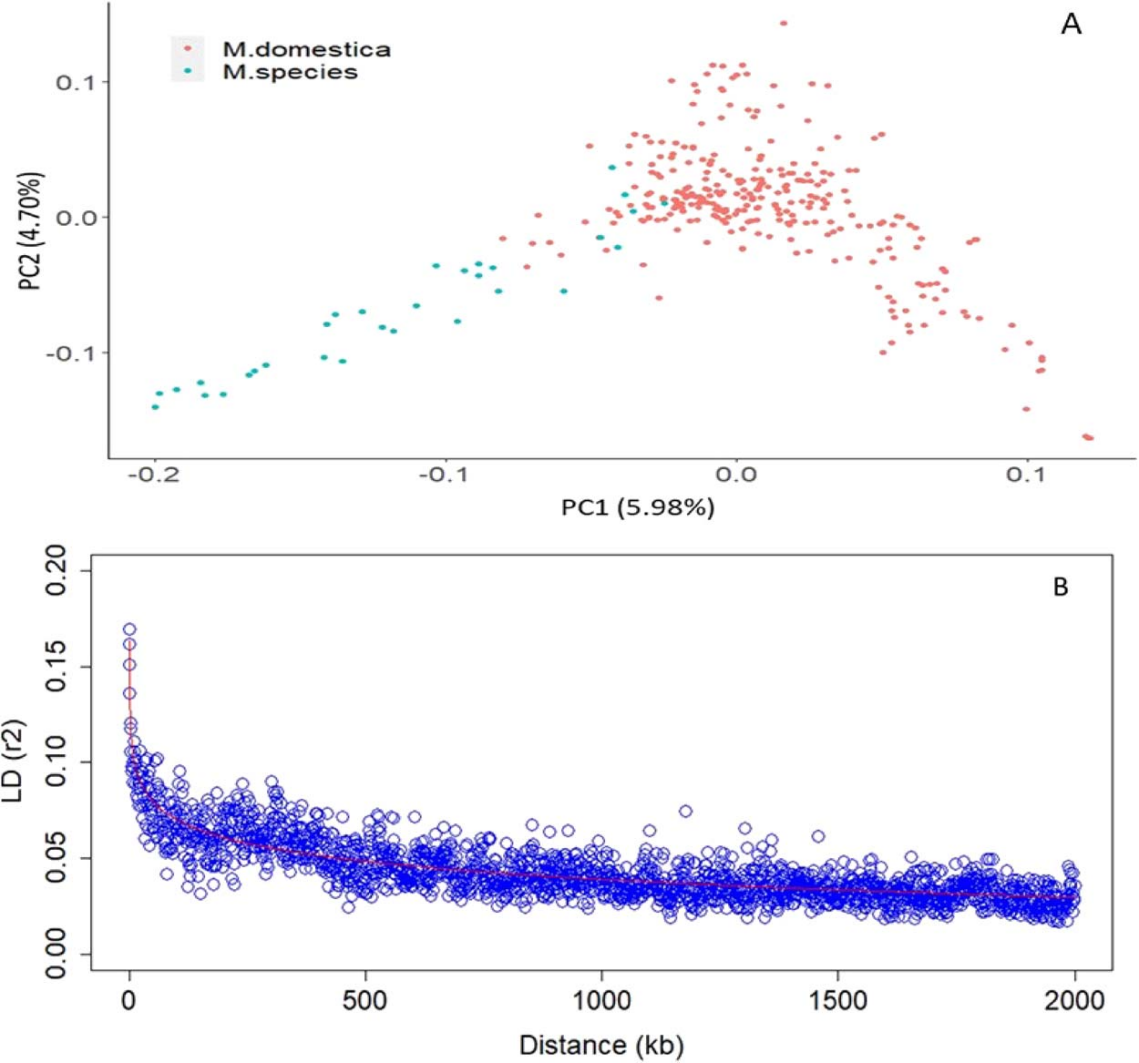
Principal components (PC) plot of 344 accessions based on their SNP profiles (A), and the plot of linkage disequilibrium (LD) decay (B).

### Genetic structure and linkage disequilibrium

The first principal component (PC1) distinguishes the accessions belonging to wild *Malus* species and *M. domestica*, while the PC2 revealed the variability within each of the two genepools (Fig. 3A). Further analysis using the Neighbour Joining (NJ) tree revealed several sub-clusters among *M. domestica* accessions (Supplementary Figure S5), which highlighted the extent of cryptic genetic relatedness among the accessions of this study. Using the admixture model, as implemented in the STRUCTURE software, the mean estimated membership of *M. domestica* individuals to the two gene pools, namely *M. domestica* and *Malus* spp., was 0.76 and 0.24, respectively. Almost all accessions of *M. domestica* showed varying degree of introgression from the wild *Malus* gene pool (Supplementary Figure S6). The average LD (*r*^2^) between SNPs separated by 100 bp was 0.17, and decayed rapidly to 0.07 and 0.05 for SNPs separated by 100 kb and 500 kb, respectively (Fig. 3B). LD decay patterns were different between the domesticated and wild genepools; for example, LD in *M. domestica* population was higher at short distances, but *Malus* spp. population displayed slightly higher LD at long distances (Supplementary Figure S9).

**Figure 4:**
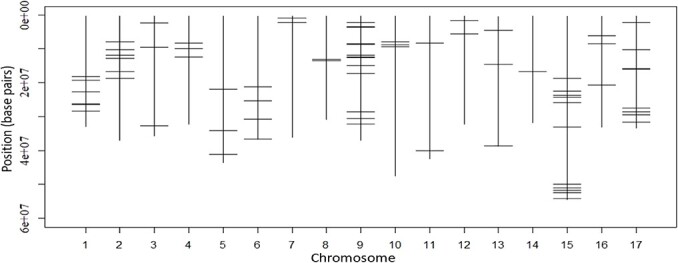
The locations of single nucleotide polymorphisms (SNPs) found to have a significant association with at least one phytochemical.

### Marker-trait associations for phytochemicals

There were multiple genomic regions significantly associated with various phytochemicals on all 17 chromosomes, and the number of significant genomic regions was the highest on chromosome 9 and the lowest on chromosomes 8 and 14 (Fig. 4; Supplementary Data Table S3). The percentage of phenotypic variance explained by the most significant SNP for each phytochemical ranged between 4% (triterpenes) and 21% (procyanidin) (Supplementary Figure S2).

### Triterpenoids

Nine triterpenoid acids (3-oxo-hydroxy-urs-12-en-28-oic acid_1, annurcoic, betulinic, corosolic, euscaphic, maslinic, pomaceic, pomolic and ursolic) were investigated in this study, but a strong association signal was found only for euscaphic acid (Supplementary Figure S7; Supplementary Data Table S3). The most significant SNPs (chr9:11985489 and chr9:12564619) flanked a cluster of xyloglucan endo-transglucosylase/hydrolase proteins (MD09G1152400 - MD09G1152700) and a galacturonosyl transferase 15 (GAUT-15: MD09G1155100) gene.

### Dihydrochalcones (DHC)

SNPs significantly associated with the three DHC compounds (3-hydroxyphloridzin, phloridzin, and phloretin-2’-*O*-xyloglucoside) were located on almost all chromosomes (Supplementary Figure S7; Supplementary Data Table S3). The glycosyltransferase protein MD01G1073400 is within 220 kb upstream of the SNP (chr1:18116719) associated with 3-hydroxyphloridzin and phloretin, while UDP-glycosyltransferase protein MdGT1 (MD01G1077200) is within 260 kb downstream. Along with a cluster of NB-ARC domain-containing disease resistance proteins (MD01G1186500, MD01G1186600, etc.), 1-amino-cyclopropane-1-carboxylate synthase 12 genes (MdACS12: MD01G1186400; MD01G1186700; MD01G1186900), and glycosyltransferase gene (MD01G1187600) also occurred close to the SNP (chr1:28379456) associated with 3-hydroxyphloridzin and phloridzin.

The most prominent association signal (chr2:10212596) for 3-hydroxyphloridzin and phloretin was about 18 kb from the 3-dehydroquinate synthase (DHQS) gene (MD02G1126200). A MYB protein (MdMYB52: MD06G1235300) is within 100 kb of the second most significant SNP (chr6: 36724439) associated with phloridzin. A SNP associated with 3-hydroxyphloridzin and phloridzin on chromosome 15 (chr15:24335063) is surrounded by a cluster of CC-NBS-LRR disease resistance proteins (MD15G1276900; MD15G1277500 etc.). A SNP at the distal end of chromosome 15 (chr15:51077031) associated with phloridzin is within 20 kb of MYB protein 3 (MdMYB3: MD15G1411200). A GWAS signal for 3-hydroxyphloridzin on chromosome 16 (chr16:8480114) occurred about 80 kb downstream of a caffeoyl-CoA 3-O-methyltransferase (CCOAMT) gene (MD16G1118200). Another significant signal for this phenotype in the upper region of chromosome 17 (chr17:2146770) occurred slightly more than 200 kb upstream of MYB24 (MD17G1032300; MD17G1032500). An association for 3-hydroxyphloridzin on chromosome 17 (chr17:10280548) is within 100 kb of a cluster of cinnamyl-alcohol dehydrogenase (CAD) genes (MD17G1119700, MD17G1119800, etc.).

A hydroxycinnamoyl-CoA shikimate/quinate hydroxycinnamoyl transferase (HCT/HQT) gene (MD17G1225100) resided about 250 kb upstream of a SNP (chr17:27602614) associated with 3-hydroxyphloridzin, and a 4-coumarate:CoA ligase (4CL) gene (MD17G1229400) was located about 140 kb downstream. In addition, at about 300 kb downstream of this SNP, there is a cluster of senescence-associated gene 12 (MD17G1231600 - MD17G1231800; MD17G1232200 - MD17G1232400).

### Flavanols

Seven compounds including flavanol monomers (catechin and epicatechin) and flavanol polymers (procyanidins B1, B2, B5, B7 and C1) were observed in this study, and the GWA signals were observed on almost all chromosomes, but the most prominent association signals were detected on chromosome 9 (Supplementary Figure S7; Supplementary Data Table S3). A SNP (chr9: 2151903) associated with all seven flavanol compounds was about 280 kb from MYB24 (MD09G1030700), while another significant SNP (chr9:3712942) was located less than 150 kb from MYB16 (MD09G1054000). MYB101 (MD09G1109900) resided within 600 kb of the SNP (chr9:8726075) associated with flavanol phenotypes. A significant signal in the central region (chr9:14903419) was located less than 30 kb from 2-oxoglutarate-dependent dioxygenase (2ODDS) family protein MD09G1177300, while a significant region at the distal end of chromosome 9 was within 10 kb of a cluster of HCT/HQT genes (MD09G1230000, MD09G1230100, etc.).

SNPs associated with one or more flavanol compounds in the upper region of chromosome 10 (Chr10:7887052 and Chr10:9437064) were flanked by a B-box zinc finger protein (MD10G1066000). A SNP (Chr2:10212596) associated with four of the seven flavanol compounds was within 20 kb of the DHQS gene MD02G1126200, and a signal in the upper region of chromosome 17 (chr17:109495) was within 18 kb of a WRKY transcription factor (WRKY75: MD17G1001500).

### Flavonols

A SNP (chr3:32741919) associated with quercetin 3-*O*-glucoside and quercetin 3-*O*-rutinoside was within 200 kb of several UGT proteins (MD03G1239700, MD03G1241300 and MD03G1241400) (Supplementary Figure S7; Supplementary Data Table S3). For quercetin 3-*O*-glucoside, a significant association on chromosome 9 (chr9:30552256) is flanked by genes involved in regulating senescence (MD09G1239100) and acyl-CoA oxidase (ACOX) (MD09G1241300). There is a polyketide synthase protein (MD17G1158100) in the vicinity of the signal on chromosome 17 (chr17:15775967). Polyketide synthase genes refer to a more general description for the *CHS* and *CHS-like* genes involved in flavonol biosynthesis.

The most significant SNPs associated with quercetin 3-*O*-rhamnoside were on chromosome 1 (chr1:26506469 and chr1:27465802), which flanked several gene functions including, a chalcone-flavanone isomerase (CHI) gene (MD01G1167300), laccase proteins (MD01G1158900 and MD01G1159400), and a cluster of disease resistance LRR family proteins (MD01G1172100; MD01G1172300; MD01G1172800). The third significant SNP for quercetin 3-*O*-rhamnoside (chr1:22717183) is within 500 kb of a chalcone-flavanone isomerase (CHI) gene cluster (MD01G1118000, MD01G1118100, etc.).

Another association signal for quercetin 3-*O*-rhamnoside in the upper region of chromosome 16 (chr16:8480114) is about 80 kb from a cluster of caffeoyl-CoA 3-*O*-methyltransferase (CCOAOMT) genes (MD16G1118200; MD16G1119200; MD16G1119300).

### Hydroxycinnamic acids

The two significant SNPs (chr2:16706831 and chr2:18670719) associated with CGA flanked the 1-aminocyclopropane-1-carboxylate synthase (ACS) gene (MD02G1190900), while the signal on chromosome 9 (chr9:17335476) is within 650 kb of a MYB domain protein 26 (MYB26: MD09G1191100) (Supplementary Figure S7; Supplementary Data Table S3). The association signals on chromosome 15 (chr15:22495262) is within 3 kb of a cluster of CC-NBS-LRR disease resistance proteins (MD15G1263100 - MD15G1263300). A CGA-associated SNP in the upper region of chromosome 16 (chr16:6141995) is flanked upstream by two UGT 74B1 proteins (MD16G1086200 and MD16G1086300) and downstream by a MYB protein (MYB66: MD16G1093200). The association signal for both hydroxycinnamic compounds (CGA and t4pCouQA) on chromosome (chr15:51077031) is within 20 kb of MYB3 (MD15G1411200).

**Figure 5:**
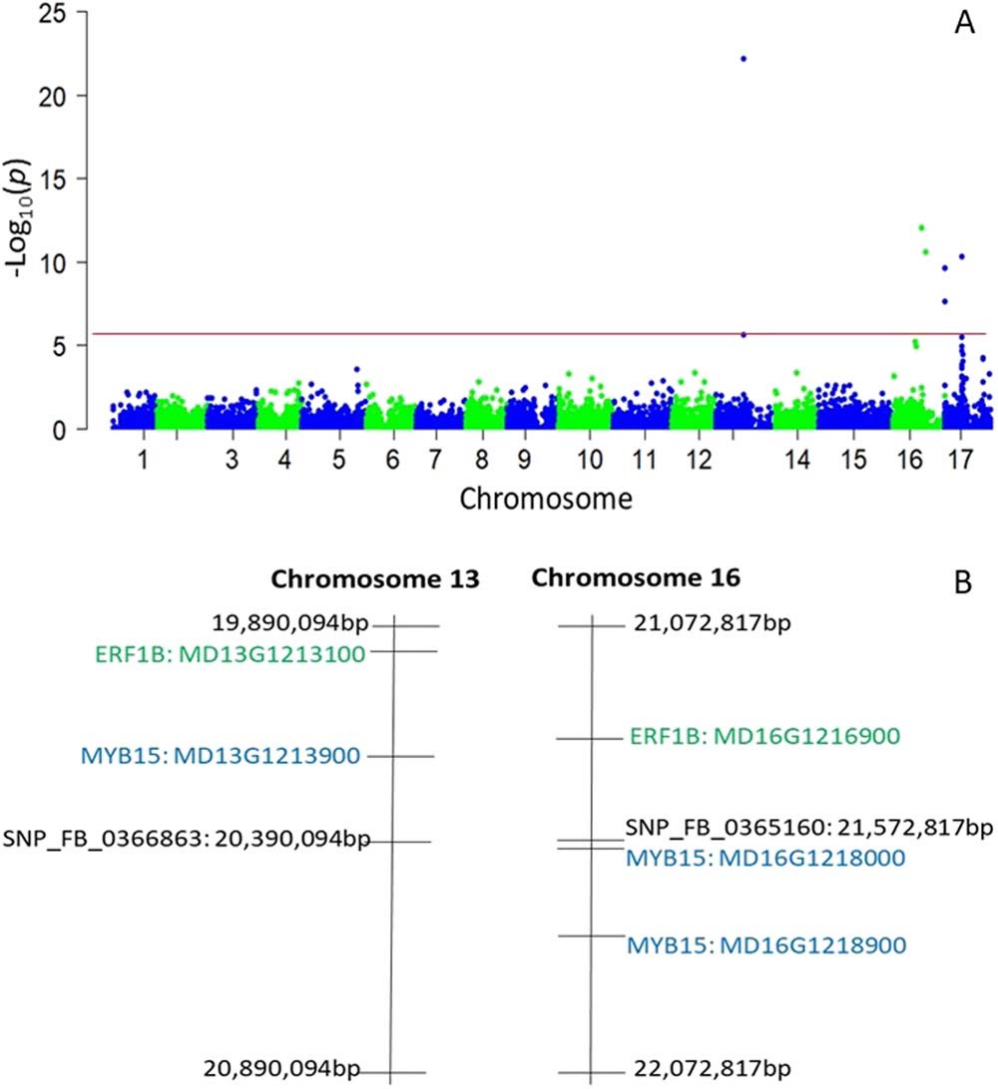
Manhattan plot of the -Log10(*p*) values for red skin colour (OCOL) from a genome-wide scan (A). The horizontal line indicates the genome-wide significance threshold. Paralogs of MYB15 and ERF1B genes in the syntenic regions on chromosome 13 and 16 (B).

### Organic acids

An association signal for MA on chromosome2 (chr2:7978884) is within 2 kb of a SAUR-like protein (MD02G1100200) and about 18 kb from pectin methylesterase 2 protein (MdPME2: MD02G1100400). Methionine sulfoxide reductase (MSR) B5 genes (MD13G1177800; MD13G1178200) were located about 80 kb downstream from a SNP (chr13:14686218) is marginally associated with AsA (Supplementary Figure S7).

### Fruit quality traits

Significant marker-trait associations were detected for five (OCOL, FBD, FIRM, CRISP, JUIC) out of 11 fruit quality traits in this study (Fig. 5; Supplementary Figure S8; Supplementary Data Table S3). There were also two chalcone synthase (CHS) genes (MD02G1218400 and MD02G1218500) within 500 kb of the SNP (chr2: 25886126) associated with flesh browning disorder (FBD). The MYB domain protein 6 (MYB6: MD09G1261200) was located about 40 kb downstream from the SNP (chr9:33373385) associated with fruit texture traits (crispness, firmness and juiciness), while a senescence-associated protein (MD09G1263800) and an S-adenosyl-L-methionine-dependent methyltransferase (SAM-MTase) protein (MD09G1258900) also resided within 300 kb. In addition, a dehydroascorbate reductase (DHAR) protein (MD09G1264700) was also located within 400 kb of this texture-associated SNP.

Three main genomic regions associated with OCOL were identified on chromosomes 13, 16 and 17 (Fig. 5). The two significant SNPs on chromosome 13 (chr13:20118900 and chr13:20390094) flanked MYB15 (MD13G1213900), while the ethylene response factor 1 (ERF1B: MD13G1213100) also resided within 150 kb upstream. Paralogs of MYB15 (MD16G1218000 and MD16G1218900) and ERF1B (MD16G1216900) underpinned the significant signal for OCOL on chromosome 16 (chr16:21572817). An association signal for OCOL in the middle region of chromosome 17 (chr17:12697927) resided within 300 kb of WRKY28 (MD17G1138100) and about 650 kb from NAC domain protein 42 (MdNAC42: MD17G1134400). SNPs significantly associated with OCOL in the upper region of chromosome 17 (285862-286 154 bp) occurred 3 kb from the acetyl-CoA synthase gene (MD17G1003300) and about 200 kb from WRKY75 (MD17G1001500).

## Discussion

### Phytochemicals variability and between-trait relationships

Similar to earlier studies [3, 12], our results revealed large variation within- and between the *M. domestica* and wild *Malus* species accessions. Compared with the other metabolite groups, differences in organic acids and DHC content between *M. domestica* and *Malus* spp. were more prominent. The fruit from wild accessions also showed significantly higher content of AsA and AA-2βG than *M. domestica* accessions.

The genomic *h*^2^ of AA-2βG was considerably higher than that of AsA (0.42 vs 0.20), and the between-season correlation for AA-2βG was also higher compared with AsA (0.95 vs 0.70). These findings would suggest that AA-2βG could be a better selection trait than AsA for improving Vitamin C content of future apple cultivars, but further research is needed on the stability of AA-2βG in the human gut and whether this will increase vitamin C bioavailability. Dihydrochalcones (e.g. phloridzin), flavanols (e.g. catechin, epicatechin) and hydroxycinnamic acids (e.g. chlorogenic acid), which are associated with several health benefits [1], tended to be slightly negatively correlated with the overall eating quality of apple fruits in this study, which would pose challenges for the simultaneous improvement of fruit quality and these metabolites.

Trilobatin has shown strong antioxidant, anti-inflammatory, and α-glucosidase inhibition activities for the management of diabetes [13]. In our study, trilobatin content was favourably correlated (~0.25) with sensory sweetness, suggesting that introgression of trilobatin in future cultivars would further enhance both health benefits and taste profiles of apples. We are conducting further studies, using segregating populations, to better understand the genetics of trilobatin content in apple fruit. The finding of a significant correlation between OCOL and ursolic acid in our study is supported by earlier reports of simultaneous production of anthocyanin and ursane/oleanane in plant cell culture [14]. However, the physiological and molecular functions underpinning the co-production of these compounds remain to be determined in apples.

### Marker-trait associations and potential candidate genes for phytochemicals

Several ripening and senescence-associated gene functions (e.g. MdEIN3: MD15G1441000, MdERF1: MD01G1177000, ACS12: MD01G1186400, MD09G1239100, MD17G1231600) were located close to the significant SNPs, supporting earlier studies showing linkage between the ripening and phytochemical biosynthesis pathways [15, 16]. The EIN3-like protein was also reported to be involved in terpene synthases in kiwifruit [15], and studies on winegrape [17] revealed a potential crosstalk between flavonol and terpenoid metabolism. This would support the occurrence of a terpenoid cyclase protein (MD11G1284300) about 75 kb from the quercetin-linked SNP.

The occurrence of several UGT proteins in close proximity to the DHC-associated regions in this study is in agreement with earlier reports on the role of UGTs that convert phloretin to phloridzin in *Malus* species [18]. The DHC-associated regions on Chr1 and Chr5 overlapped with previous studies [7–9]. Chalcone-flavanone isomerase (CHI) is one of the enzymes involved in the quercetin biosynthesis pathway, and several CHI genes (MD01G1118000, MD01G1118100, etc.) were located in the region associated with quercetin glycosides on chromosome 1, and this region overlaps with a previously published QTL [7, 8]. Overexpression of CHI genes was shown to increase flavonols in “Royal Gala” fruit [19].

This is the first study to identify the upper region (1.80–3.60 Mb) of Chr9 as a major hotspot for flavanol regulation. This region harbours MYB transcription factors MYB24 (MD09G1030700) and MYB16 (MD09G1054000). Transgenic assays showed that MYB16 affects the regulation of anthocyanin and flavanol in apples [20, 21], and this gene has also been reported as a major regulator of cuticle formation in apples [22]. MYB24 was reported to promote flavonoid biosynthesis through regulation of *FLS1* gene expression in *Arabidopsis thaliana* [23]*,* and MYB24 also plays a role in flavonoid regulation in grape berries [24]. *The MYB transcription factor MYB101 (*MD09G1109900), underpinning a significant region, was shown to play role in chalcone synthase (CHS) leading to flavonoid biosynthesis in *Lotus japonicus* [25] and *Rosa rugosa* [26].

The occurrence of MYB52 (MD06G1235300) and MYB3 (MD15G1411200) within genomic hotspots for phloridzin and CGA is supported by previous reports. For example, overexpression of MdMYB3 in tobacco resulted in upregulation of several flavonoid pathway genes, including CHS, CHI, UFGT and FLS [27]. The expression of MYB52 was reported higher, along with the content of phloridzin, in russeted apples, suggesting a potential indirect role of this drought-stress response gene in phloridzin biosynthesis [22]. MYB26 (MD09G1191100), underpinning the GWAS signals for CGA on chromosome 9, was shown to be involved in the regulation of phenylpropanoid production in pea [28].

The HCT/HQT and 4CL genes (MD17G1225100 and MD17G1229400) resided in the DHC (3-hydroxyphloridzin) associated genomic regions, and these gene functions were hypothesised to play a role in the regulation of CGA [9] and phloridzin [29]. HCT/HQT are also reportedly linked to chalcone and flavonoid synthesis in plants [30], and a cluster of HCT/HQT proteins flanked a flavanol-associated signal at the distal end of Chr9 which is paralogous to the DHC-associated region on Chr17.

Our GWA results suggest that a CCOAMT gene (MD16G1118200), CAD genes (MD17G1119700; MD17G1119800) and DHQS gene (MD02G1126200) are potential candidates underpinning DHC-associated genomic regions. These gene functions were also proposed as candidate genes for the biosynthesis of hydroxycinnamic acids (CGA) [9], which is supported by the high genetic correlation (ca. 0.65) between phloridzin and CGA in this study. The CCOAMT enzyme was reported to play a key role in the synthesis of quercetin in citrus [31], which gives support to the occurrence of a cluster of CCOAMT genes in the vicinity of a quercetin-associated SNP in the upper region of chromosome 16 (chr16:8480114) in this study.

Overexpression of B-box (BBX) genes in apple callus led to the higher expression of anthocyanin biosynthesis-related genes (*MdMYB1, MdANS*) and flavonoid pathway genes (*MdCHS*, *MdF3H* and *MdDFR*) [32], which support the presence of a BBX protein (MD10G10660000) in the flavanol-associated region on Chr10 in our study. A significant signal for flavanol compounds in the central region of chromosome 9 was underpinned by 2ODDS gene (MD09G1177300), and this family of gene systems was shown to be involved in the flavonoid biosynthesis pathway in tobacco [33].

Clusters of 2-oxoglutarate (2OG) and Fe(II)-dependent oxygenase proteins occurred within the flavonols-linked regions on Chr1 and Chr16. 2OG genes are involved in the synthesis of anthocyanin in crabapples [34], and it was shown that overexpression of anthocyanin biosynthesis genes promoted transcription of some flavonoid biosynthetic genes and induced quercetin accumulation in crabapples [21]. A study on *Arabidopsis thaliana* also reported that flavonol synthase requires 2OG and ferrous iron (Fe) for catalytic function [35]. The concentration of flavonols was reported negatively correlated with laccase activity in apple cultivars [36], and interestingly the flavonols-associated hotspot on Chr1 harbours two laccase proteins (MD01G1158900 and MD01G1159400) in our study.

### Putative candidate genes for fruit quality traits

We identified a novel texture-associated signal on Chr9 in this study, and the MYB6 (MD09G1261200), located within 50 kb of the significant SNP, has been shown to play a role in ripening and storability of strawberry [37]. Some other gene functions residing in this region include a senescence-associated protein (MD09G1263800), the S-adenosyl-L-methionine-dependent methyltransferases (SAM-MTase) protein (MD09G1258900), and a dehydroascorbate reductase (DHAR) protein (MD09G1264700). SAM-MTase acts in the biosynthesis pathway of ethylene and polyamines, and plays important roles during the ripening process and may affect fruit firmness [38]. A DHAR protein, known for its role in limiting oxidative stress, was shown to be upregulated in highly firm apple fruit, suggesting a role in maintaining fruit texture [39].

Our study identified novel genomic regions associated with OCOL variation in apple germplasm. Among other gene functions, paralogs of MYB15 and ERF1B underpinned the association signals for OCOL in the syntenic regions on Chr13 and Chr16 – suggesting that whole genome duplication has played a role in the evolution of apple red skin colour. The levels of anthocyanin significantly increased in MdERF1B-overexpressing callus [40, 41], while the expression of MYB15 was shown to be upregulated in “Royal Gala” skin under hot conditions, suppressing the regulation of anthocyanin [20]. Further studies are needed to understand the interplay between these two contrasting anthocyanin regulatory genes ERF1B and MYB15.

There were two WRKY transcription factors (WRKY75: MD17G1001500; WRKY28: MD17G1138100) in the OCOL-associated regions on Chr17. Both of these WRKY transcription factors have been reported being involved in the regulation of anthocyanin biosynthesis [42, 43]. It has been suggested that the acetyl-CoA pool could be directed to anthocyanin biosynthesis during many different stresses [44], which supports the occurrence of the acetyl-CoA synthase gene (MD17G1003300) within 3 kb of the OCOL-associated SNPs in the upper region of Chr17. NAC42, supporting the OCOL-associated region in the middle of Chr17, regulates anthocyanin accumulation in apple by interacting with MYB10 [45]. Our results suggest that the genetic regulation of apple red skin is complex and potentially controlled by the interplay between several gene families.

### Breeding for phytochemicals

The content of several key phytochemicals was significantly higher in wild apple accessions, suggesting that these accessions could be used as parents for introgression breeding to develop phytonutrient-rich apple cultivars. In this germplasm study, the correlations between eating quality traits and phytochemicals were moderate, which suggested that it would be feasible to develop tailored breeding strategies for the simultaneous improvement of these two groups of traits. Studies using segregating populations would be helpful to further understand the genetic correlations between eating quality and phytochemicals, which would guide future cultivar development strategies. The complex polygenic architecture of most metabolites in this study suggested that genomic selection would be a better option to reduce the generation interval for introgression breeding [46].

## Materials and methods

### Plant material

The ex situ *Malus* germplasm collection maintained at the Hawke’s Bay site (39°40’S 176°53′E) of The New Zealand Institute for Plant and Food Research Limited was established as grafted trees in late 1950s when budwood from heritage cultivars and crab-apples was imported mainly from the United Kingdom. In 2013, each accession was re-propagated onto “M9” rootstock and maintained in the orchard following standard commercial practices. This study was performed on 344 accessions, comprising 313 *M. domestica* accessions and 31 accessions of wild *Malus* species (Supplementary Data Table S1).

### Metabolite phenotyping

Metabolite phenotyping was conducted over a period of two fruiting seasons. Of the 344 accessions, 165 and 179 were assessed in 2019 and 2020, respectively. There were 15 accessions common between the two years to estimate seasonal effect and obtain seasonally adjusted phenotypes of all accessions.

### Sample preparation

We followed fruit harvesting and storage protocols as reported earlier by Kumar *et al*. [47]. Briefly, the fruit were stored for four weeks at 0.5°C, then a further 1 day at 20°C before evaluation. For each accession, a total of 16 cores were cut from four apples at four positions around the equatorial circumference of each apple using a #5 corer (9 mm internal diameter). Each core contained apple skin and cortex (but no apple core or seeds) and was trimmed to approximately 1 cm if required. The total weight of the 16 cores was approximately 10 g and the actual weight was recorded (two decimal places). The 16 apple cores were added to a 250 mL Schott bottle containing 100 mL solvent (ethanol:water:formic acid 80:20:2 with the internal standard naringenin at 1.05 mg/L) and homogenized using the Omni General Laboratory Homogenizer (GLH850, Kennesaw, GA, USA). Samples were left overnight at 4°C to extract and then an aliquot was transferred to a centrifuge tube for analysis.

### Analysis of ascorbic acid (AsA) and ascorbic acid 2-β-glucoside (AA-2βG) by UHPLC

Immediately after extraction, a portion of the extract was diluted 1:1 with 2 mM tris(2-carboxyethyl) phosphine hydrochloride (TCEP.HCl) (Sigma-Aldrich, Sydney, Australia) in MeCN/formic acid/water (90:5:5) to preserve the AsA. The actual volume used was 200 μL extract +200 μL 2 mM TCEP in MeCN/formic acid/water (90:5:5) directly into LC vials. The UHPLC system Dionex Ultimate® 3000 Rapid Separation LC was composed of a pump, autosampler, column oven, and Photodiode Array (PDA) detector. The LC column was a Luna HILIC 150 mm x 4.6 mm, 3 μm (Phenomenex) and was maintained at 40°C. The flow was 1000 μL min^−1^. The solvents were A = 20 mM HCOONH_4_, pH = 5.0 and B = 100% acetonitrile. The solvent composition was isocratic at 20% A and 80% B. The run time for each analysis was 10 min. The injection volume for samples and standards was 3 μL. Data were processed using Chromeleon (ThermoScientific). The response was calibrated using AsA and AA-2*α*G, therefore AA-2βG was quantified as AA-2*α*G equivalents. It is noteworthy that AsA is unstable and can degrade during the analytical process. The sample preparation methods were not optimised specifically for AsA, so the measurements of AsA in this study are estimates only. In contrast, AA-2βG is a very stable form of Vitamin C [5], and reliable quantitative results were obtained.

### Analysis of polyphenolic metabolites by LC-HRAM-MS

Each sample extract was diluted 1:1 with methanol (actual volumes: 200 μL extract +200 μL methanol) and then analysed for polyphenols by Liquid Chromatography – High Resolution Accurate Mass – Mass Spectrometry (LC-HRAM-MS) as described earlier [5]. LC–MS grade acetonitrile (Supelco, Bellefonte, PA, USA), ethanol, methanol and formic acid were purchased from Thermo Fisher Scientific (Waltham, MA, USA). Ultrapure water was obtained from a Milli-Q Intergral3 system (Millipore, Burlington, MA, USA). The LC column was a Luna Omega C18 100x2.1 mm, 1.6 μm (Phenomenex, Torrance, CA, USA) and was maintained at 40°C. The flow was 400 μL min^−1^. The solvents were A = 0.2% formic acid and B = 100% acetonitrile. The solvent gradient was: initial composition 90% A 10% B, 0–0.5 min; linear gradient to 60% A 40% B, 0.5–7 min; linear gradient to 5% A 95% B, 5–12 min; composition held at 5% A 95% B, 12–15 min; linear gradient to 90% A 10% B, 15–15.2 min; to return to the initial conditions before another sample injection at 18 min. The injection volume for samples and standards was 1 μL. The micrOTOF QII parameters were same as those described earlier [5]. Data were processed using TASQ (Bruker Daltonics, Bremen, Germany). The chemical data for the metabolites measured in this study are presented in Supplementary Table S2.

### Fruit quality traits

Thin wedges of cortical tissue were cut from each of the two apples from each accession for sensory evaluation by one trained assessor. Following Kumar *et al*. [47], several sensory traits including, firmness (FIRM), crispness (CRISP), juiciness (JUIC), sweetness (SWET), sourness (SOUR), astringency (ASTR), flavour intensity (FINT) and overall eating quality (EQUA) were assessed on a scale from 0 (= lowest) to 9 (= highest). Red skin over-colour (OCOL) coverage was scored visually on a scale 0 (no red colour) to 9 (completely red). Average fruit weight (AVFW) was measured as the average weight (g) of the six fruits per accession. The flesh browning disorder (FBD) was also recorded as reported earlier by Kumar *et al*. [51]. As fruit of some *Malus* species accessions were very small and/or had an unpleasant taste, such accessions were assessed only for AVFW and OCOL.

### SNP array genotyping and quality control

Leaf DNA from all 344 accessions were genotyped using the 20 K Infinium SNP array [48], and SNP calling was conducted using Genome Studio (Illumina Inc., San Diego, CA, USA). SNPs with minor allele frequency (MAF) <0.02, and missing data frequency > 10% were discarded, and the missing genotypes at the retained SNPs were imputed using k-nearest neighbour method. The coordinates of the filtered SNPs were identified on the GDDH13 genome assembly [49] by blasting their flanking sequences to the GDDH13 genome and detaining the best hits with the aligned length matching the flanking sequence length and having ≥90% identity.

### Data analysis

Exploratory analyses, including trait distribution, correlation between traits, and principal component analysis (PCA) were conducted using the R software (R Core Team, 2019). The two-sample *t*-test was used to test whether the population means of two groups (*M. domestica* and *Malus* spp.) are equal or not. The genetic structure of the 344 accessions was estimated using PCA, Neighbour Joining (NJ) tree, and model-based Bayesian clustering using ADMIXTURE software. Estimates of linkage disequilibrium (LD: *r*^2^) were calculated between SNPs located on the same linkage group (LG) using R package “LDcorSV”. The estimates of pair-wise LD between SNPs were averaged in the increments of one kilobase (kb) distance, and the LD decay curve was fitted using a standard logarithmic function.

The best linear unbiased estimates (BLUEs), adjusted for year effect, were used as phenotypes for GWAS. A mixed linear model (MLM) approach, accounting for cryptic relatedness (K-matrix) and population stratification (Q-matrix), implemented in R package GAPIT [50], was used for genotype–phenotype association analyses. A genome-wide significance threshold of *α* = 0.01 was used to identify significant genotype–phenotype associations. The putative candidate genes within 500 kb upstream and downstream of the most significant SNPs were retrieved using GDDH13 genome v.1.1 Jbrowser (https://www.rosaceae.org/jbrowse/). Genomic best linear unbiased prediction (GBLUP) of the additive genetic value of all accessions were obtained for each trait, and then the product–moment correlations between GBLUP for different traits were used as estimates of genetic correlations. Following Kumar *et al*. [51], the estimates of genomic heritability (*h*^2^) were also calculated for each trait using R package GAPIT.

## Supplementary Material

Web_Material_uhac218

## Data Availability

The data supporting the findings of this study are available from the corresponding author upon reasonable request.
