# Supplementary material for: GWAS provides new insights into the genetic mechanisms of phytochemicals production and red skin colour in apple
Source: Hortic Res. 2022 Sep 26;9:uhac218. doi: 10.1093/hr/uhac218 (PMC9720448; doi:10.1093/hr/uhac218)
Supplement: Web_Material_uhac218 [file web_material_uhac218.zip › Supplementary Data Table S2.docx]

Supplementary Table S2. Metabolite information: chemical data for the metabolites measured in this study.

| **Metabolite Name** | **CAS** | **Formula** | **Confidence^A^** | **RT(min)^B^** | **Quantitation^C^** |
| --- | --- | --- | --- | --- | --- |
| 3-hydroxyphloridzin | 30779-02-3 | C21H24O11 | 2 | 4.75 | phloridzin |
| 3-oxo-hydroxy-urs-12-en-28-oic acid_1 |  | C30H46O4 | 3 | 12.08 | annurcoic acid |
| annurcoic acid | 877995-09-0 | C30H46O5 | 2 | 10.89 | annurcoic acid |
| ascorbyl 2-beta-glucoside | 562043-82-7 | C12H18O11 | 1 | - | ascorbyl 2-alpha-glucoside |
| ascorbic acid | 50-81-7 | C6H8O6 | 1 | - | ascorbic acid |
| betulinic acid | 472-15-1 | C30H48O3 | 1 | 12.46 | annurcoic acid |
| catechin | 154-24-4 | C15H14O6 | 1 | 2.22 | catechin |
| chlorogenic acid | 327-97-9 | C16H18O9 | 1 | 2.27 | chlorogenic acid |
| cis-4-p-coumaroyl quinic acid | 934391-78-3 | C16H18O8 | 3 | 2.99 | trans-4-p-coumaroyl quinic acid |
| corosolic acid | 4547-24-4 | C30H48O4 | 2 | 11.34 | annurcoic acid |
| epicatechin | 490-49-0 | C15H14O6 | 1 | 3.01 | epicatechin |
| euscaphic acid | 53155-25-2 | C30H48O5 | 2 | 10.07 | annurcoic acid |
| linoleic acid | 60-33-3 | C18H32O2 | 1 | 13.6 | linoleic acid |
| malic acid | 6915-15-7 | C4H6O5 | 1 | 0.6 | malic acid |
| maslinic acid | 4373-41-5 | C30H48O4 | 2 | 11.27 | annurcoic acid |
| oleic acid | 112-80-1 | C18H34O2 | 1 | 14.39 | linoleic acid |
| phloretin-2'-O-xyloglucoside | 145758-09-4 | C26H32O14 | 2 | 4.82 | phloridzin |
| phloridzin | 60-81-1 | C21H24O10 | 1 | 5.39 | phloridzin |
| pomaceic acid |  | C30H46O6 | 2 | 9.88 | annurcoic acid |
| pomolic acid | 13849-91-7 | C30H48O4 | 2 | 11.17 | annurcoic acid |
| procyanidin B1 | 20315-25-7 | C30H26O12 | 1 | 1.65 | procyanidin B2 |
| procyanidin B2 | 29106-49-8 | C30H26O12 | 1 | 2.67 | procyanidin B2 |
| procyanidin B5 | 12798-57-1 | C30H26O12 | 2 | 3.49 | procyanidin B2 |
| procyanidin B7 | 12798-59-3 | C30H26O12 | 2 | 4.23 | procyanidin B2 |
| procyanidin C1 | 37064-30-5 | C45H38O18 | 2 | 3.28 | procyanidin B2 |
| quercetin 3-arabinopyranoside | 22255-13-6 | C20H18O11 | 2 | 4.83 | quercetin 3-rutinoside |
| quercetin 3-galactoside | 482-36-0 | C21H20O12 | 1 | 4.3 | quercetin 3-rutinoside |
| quercetin 3-glucoside | 482-35-9 | C21H20O12 | 1 | 4.4 | quercetin 3-rutinoside |
| quercetin 3-rhamnoside | 522-12-3 | C21H20O11 | 1 | 4.98 | quercetin 3-rutinoside |
| quercetin 3-rutinoside | 153-18-4 | C27H30O16 | 1 | 4.16 | quercetin 3-rutinoside |
| quercetin 3-xyloside | 549-32-6 | C20H18O11 | 2 | 4.62 | quercetin 3-rutinoside |
| trans-4-p-coumaroyl quinic acid | 1108200-72-1 | C16H18O8 | 1 | 3.15 | trans-4-p-coumaroyl quinic acid |
| trans-5-p-coumaroyl quinic acid | 5746-55-4 | C16H18O8 | 2 | 3.67 | trans-4-p-coumaroyl quinic acid |
| trilobatin | 4192-90-9 | C21H24O10 | 1 | 5.83 | phloridzin |
| ursolic acid | 77-52-1 | C30H48O3 | 1 | 12.61 | annurcoic acid |

**Notes:**

A: Confidence of the chemical identification for each metabolite reported; high to low (1-3) confidence 1 = confirmed, metabolite data corresponds to a known and validated analytical standard; 2 = probably, multiple evidences (e.g. accurate mass, fragmentation spectra, previously reported for apple); 3 = tentative, less evidence, or the exact chemical structure cannot be reliably described.

B: Metabolite retention times observed using the LC-HRAM-MS system described in the Materials and Methods section.

C: Quantitation equivalence. Analytical standards are not available for all metabolites and surrogate calibration curves of a chemically similar metabolite were used for quantitation for these metabolites. For example, all the procyanidin metabolites were quantified as procyanidin B2 equivalents.
