# Supplementary material for: GWAS provides new insights into the genetic mechanisms of phytochemicals production and red skin colour in apple
Source: Hortic Res. 2022 Sep 26;9:uhac218. doi: 10.1093/hr/uhac218 (PMC9720448; doi:10.1093/hr/uhac218)

**Supplementary Figure S1.** Range and distribution of the concentrations of phytochemicals across 344 accessions. The lower and upper hinges of the boxplots correspond to the 25^th^ and 75^th^ percentiles, respectively.

**Supplementary Figure S2.** Genomic heritability (*h*^2^) estimates for phytochemical and fruit quality traits. The percentage of phenotypic variance explained by the best SNP for each trait is also shown to the right of each bar.


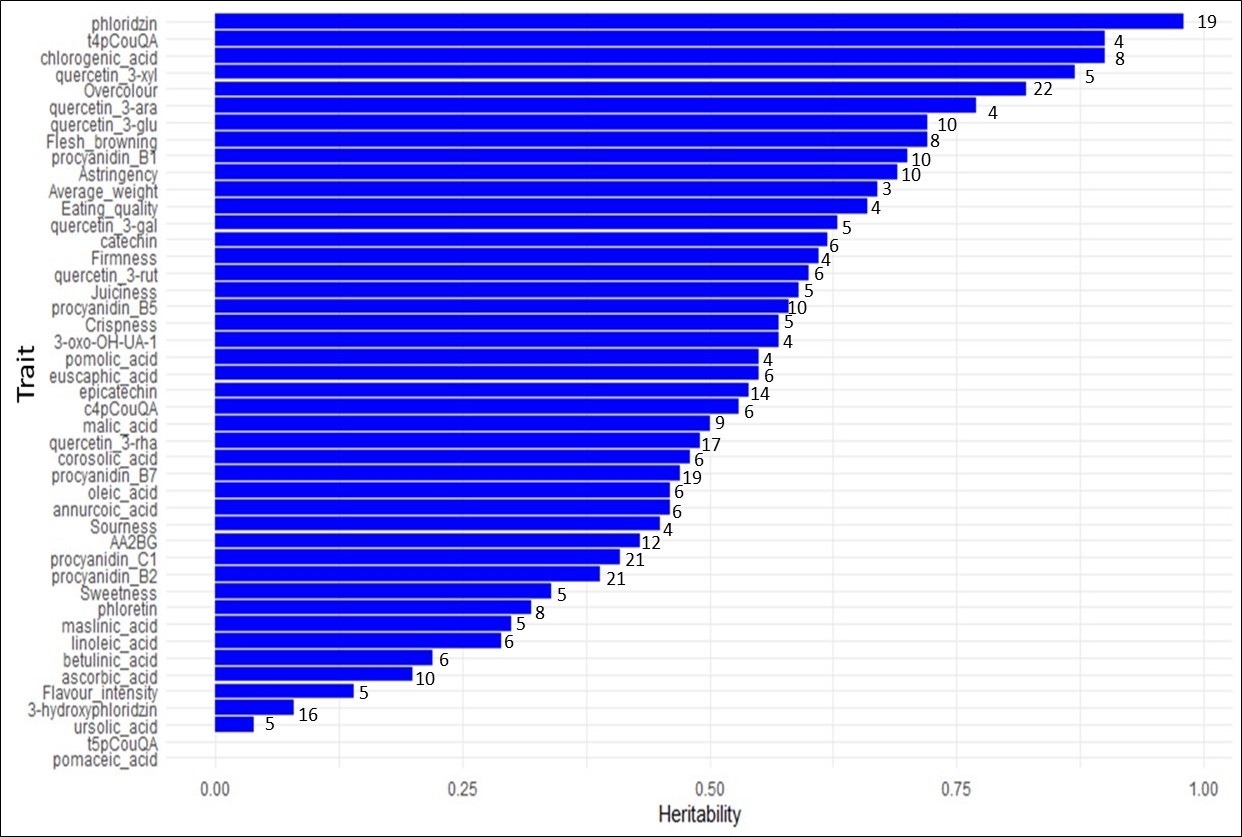


**Supplementary Figure S3.** The degree and the significance (* p < 0.01, ** p < 0.001) of correlations among pairs of phytochemicals measured on 344 accessions.


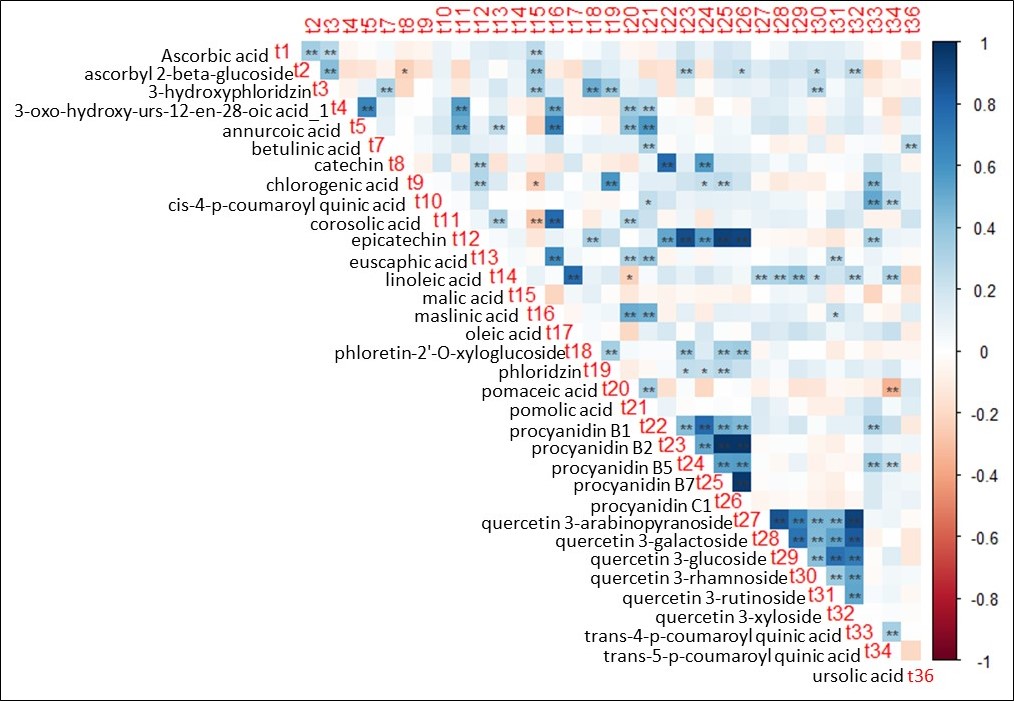


**Supplementary Figure S4.** Heat map showing genetic correlations between phytochemicals and fruit quality traits.


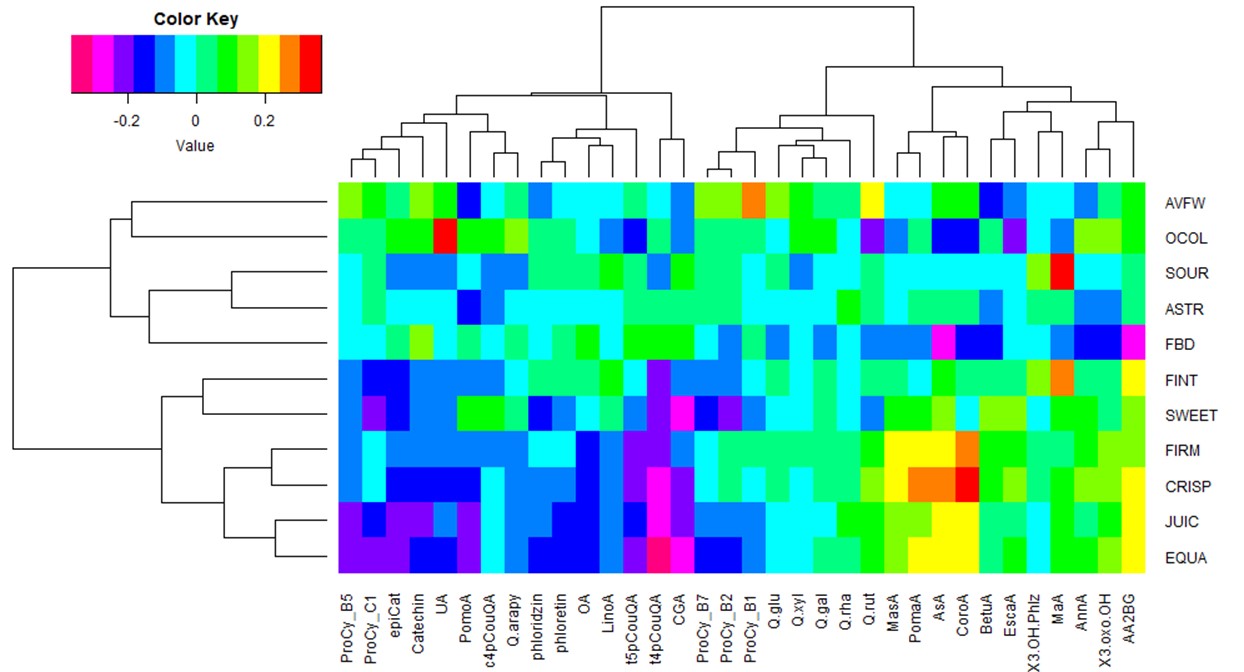


**Supplementary Figure S6.** Model-based Bayesian clustering of 344 accessions of the two gene pools (*Malus domestica* and *Malus* spp.) using the STRUCTURE software. Each accession’s genome is represented by a single vertical line, which is partitioned into coloured segments in proportion to the estimated membership of a cluster. Results shown are for varying numbers of clusters (i.e. K = 2 or 3), so the y-axis indicates the posterior probability for assignment to different clusters.


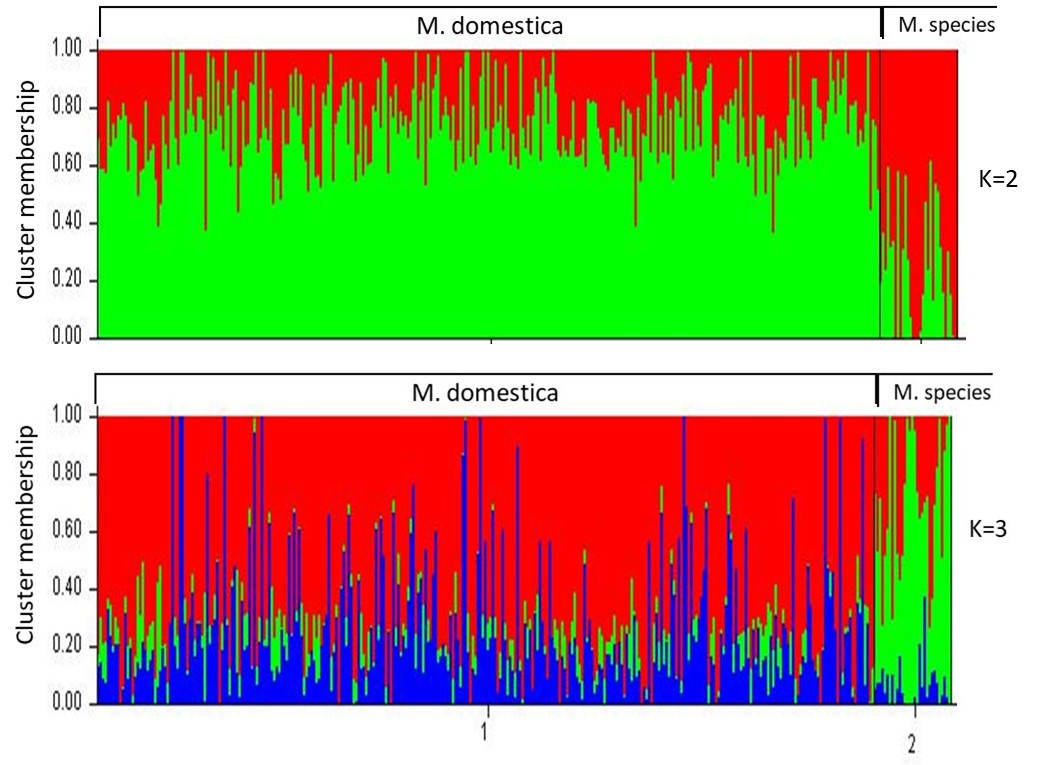


**Supplementary Figure S7.** Manhattan plot of GWAS for the individual phytochemical traits, and also for the first principal component (PC1) derived from all phytochemical traits.

**Supplementary Figure S7.** Continued.

**Supplementary Figure S8.** Manhattan plot of FQ traits (AVFW: fruit weight; EQAU: eating quality; SWEET: sweetness; FINT: flavour intensity; ASTR: astringency; FBD: internal fruit browning disorder; FIRM: firmness; CRISP: crispness; JUIC: juiciness; SOUR: sourness).


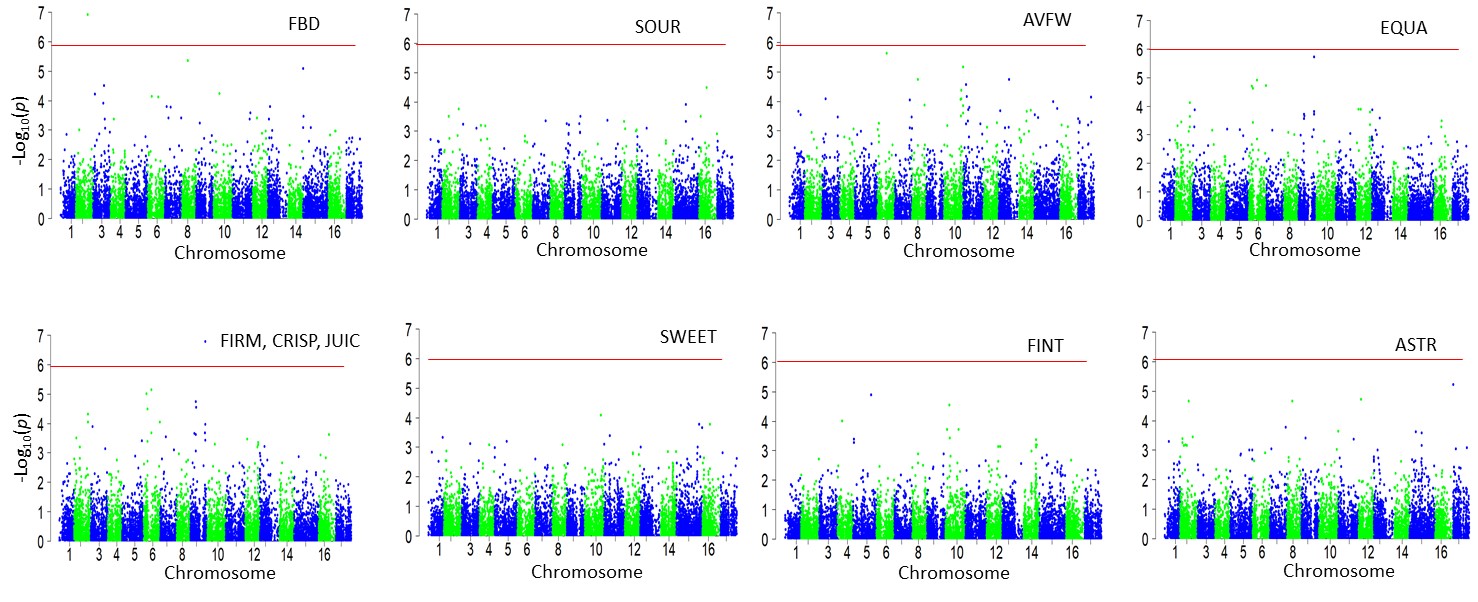


**Supplementary Figure S9**. Patterns of linkage disequilibrium (LD: *r*^2^) in *M. domestica* and *Malus* spp. genepools.


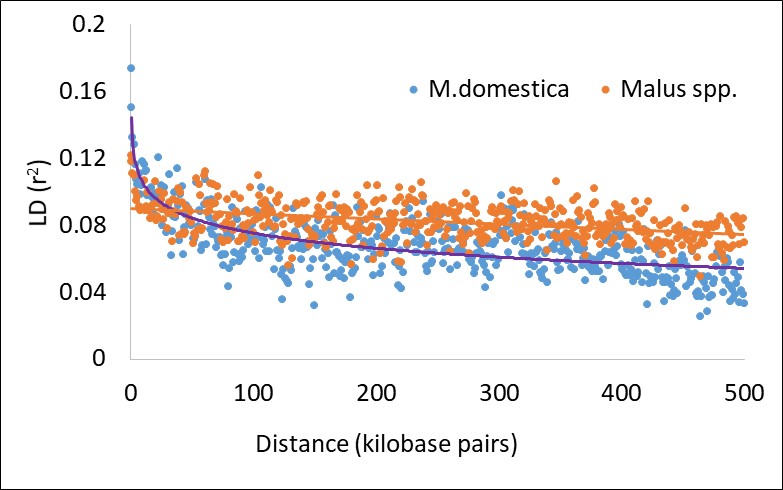

Supplement: Web_Material_uhac218 [file web_material_uhac218.zip › Supplementary Figures 1 2 3 4 6 7 8 9_R1.docx]
