# Supplementary figures and images for: GWAS provides new insights into the genetic mechanisms of phytochemicals production and red skin colour in apple
Source: Hortic Res. 2022 Sep 26;9:uhac218. doi: 10.1093/hr/uhac218 (PMC9720448; doi:10.1093/hr/uhac218)

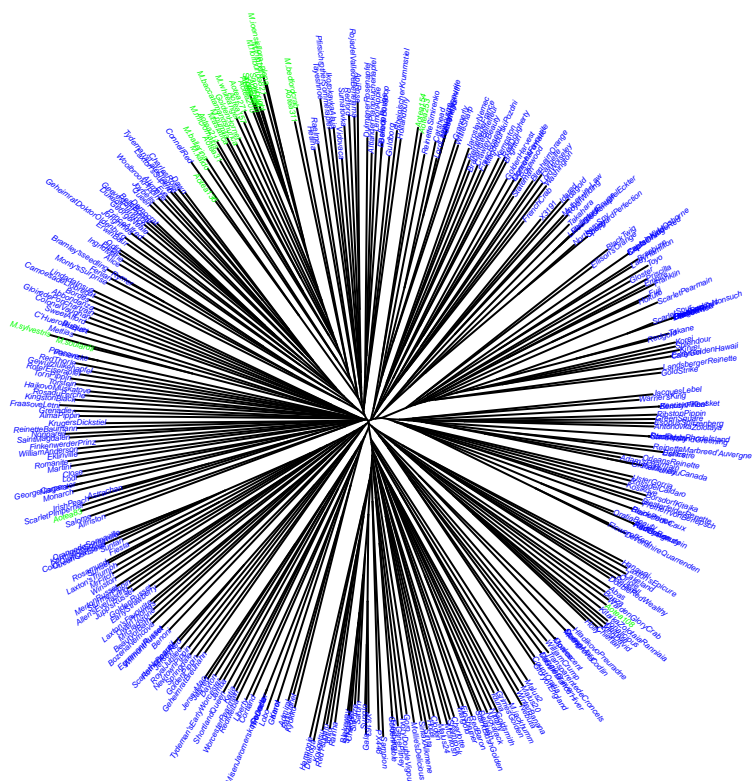

Supplement: Web_Material_uhac218 [file web_material_uhac218.zip › Supplementary Figure S5_NJ.pdf]
